# Sex-Related Effects of Gut Microbiota in Metabolic Syndrome-Related Diabetic Retinopathy

**DOI:** 10.3390/microorganisms11020447

**Published:** 2023-02-10

**Authors:** Andrea García-Llorca, Georgios Kararigas

**Affiliations:** Department of Physiology, Faculty of Medicine, University of Iceland, 101 Reykjavík, Iceland

**Keywords:** biological sex, biomarkers, cardiovascular disease, diabetic retinopathy, gut microbiota, metabolic syndrome

## Abstract

The metabolic syndrome (MetS) is a complex disease of metabolic abnormalities, including obesity, insulin resistance, hypertension and dyslipidaemia, and it is associated with an increased risk of cardiovascular disease (CVD). Diabetic retinopathy (DR) is the leading cause of vision loss among working-aged adults around the world and is the most frequent complication in type 2 diabetic (T2D) patients. The gut microbiota are a complex ecosystem made up of more than 100 trillion of microbial cells and their composition and diversity have been identified as potential risk factors for the development of several metabolic disorders, including MetS, T2D, DR and CVD. Biomarkers are used to monitor or analyse biological processes, therapeutic responses, as well as for the early detection of pathogenic disorders. Here, we discuss molecular mechanisms underlying MetS, the effects of biological sex in MetS-related DR and gut microbiota, as well as the latest advances in biomarker research in the field. We conclude that sex may play an important role in gut microbiota influencing MetS-related DR.

## 1. Introduction

The metabolic syndrome (MetS) is associated with a cluster of comorbidities, including obesity, insulin resistance, hypertension and dyslipidaemia, and it is closely linked to an increase of atherosclerotic cardiovascular disease in individuals with type 2 diabetes (T2D), as well as in non-diabetics [1,2,3]. Early diagnosis is crucial in order to successfully implement lifestyle and risk factor modification. Globally, the prevalence of MetS varies and frequently relates to the incidence of obesity [4,5]. There is a major influence of race/ethnicity, age and biological sex. About a quarter of people in Europe are affected by MetS [2]. In the USA, the prevalence is about 32.1% among obese teenagers [6]. Although south-east Asia has a lower prevalence of MetS, it is quickly catching up to rates seen in the western world. It has been reported that a higher socio-economic status is a protective factor, probably starting in early adolescence, while its positive effect may eventually wane with advancing age [7]. Lower incomes and levels of education are related with a higher prevalence of MetS in both men and women, while this correlation is significantly greater in women than in men [8,9,10]. According to recent data, MetS prevalence is often higher in men than women, but only up until the age of 50, at which point there is a shift in the opposite direction [11]. In addition, postmenopausal women are more susceptible to develop MetS and experience more cardiovascular events compared with men of the same age [12]. After menopause, a woman’s chance of developing MetS and insulin resistance rises, which is correlated with a drop in high-density lipoproteins and an increase in circulating triglycerides and low-density lipoproteins [13]. In women with T2D, hormone therapy (HT) has been linked to positive effects on glycaemic management [14].

Insulin resistance is defined as a compromised physiologic response of target tissues, predominantly the muscle, liver and adipose tissue, to insulin stimulation. Hyperinsulinemia and increase in beta-cell insulin synthesis occur as a result of impaired glucose elimination. Hyperglycaemia, dyslipidaemia, hypertension, obesity, hyperuricemia, increased inflammatory markers, endothelial dysfunction and a prothrombic state are some of the metabolic consequences of insulin resistance. T2D and MetS can develop as a result of the progression of insulin resistance [4,15]. Uncontrolled T2D may increase the chance of developing diabetic complications. Immune dysfunction and microvascular diseases are frequent diabetic complications, ultimately leading to several pathologies, including nephropathy, neuropathy, diabetic ulcer disease, cerebrovascular episodes, cardiovascular disease (CVD) and retinopathy [16,17,18]. Among these pathologies, diabetic retinopathy (DR) is a common microvascular complication [19]. Gut microbiota have recently emerged as a crucial component in both health and disease. In particular, gut microbiota are expected to have a major impact on MetS, T2D, as well as CVD, which could be in a sex-biased manner [20,21]. In fact, the association between MetS-related vascular complications and gut microbiota is demonstrated in several studies assessing vascular stiffness [22]. Here, we discuss molecular mechanisms underlying MetS, the effects of biological sex in MetS-related DR and gut microbiota, as well as the latest advances in biomarker research in the field.

## 2. Overview of Altered Molecular Mechanisms in Metabolic Syndrome

**Figure 1:**
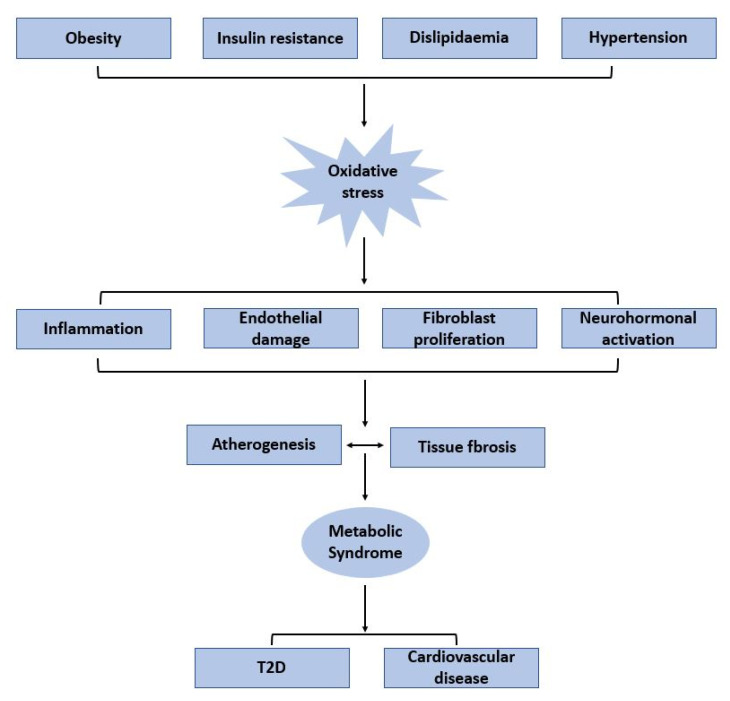
Key players in the pathogenesis and complications of metabolic syndrome.

The players that appear important in the pathophysiology of MetS and its complications are inflammation, neurohormonal activation and insulin resistance. In addition, both obesity and insulin resistance induce systemic oxidative stress, which, in turn, increases the activation of downstream signalling cascades that result in atherogenesis and tissue fibrosis (Figure 1). Tumour necrosis factor α (TNF-α), the most extensively studied cytokine, plays a crucial role in the modulation of insulin resistance seen in obesity and T2D [23]. Increased blood TNF-α levels are linked to obesity and insulin resistance, both of which are major contributors of MetS [24]. The metabolic effects of obesity are caused by the action of interleukin 6 (IL-6), a cytokine released by adipocytes and macrophages. In the liver, endothelium and bone marrow, IL-6 leads to the production of acute phase reactants, particularly c-reactive protein (CRP). It has been reported that there is a clear association between high levels of CRP and cardiac events, T2D and MetS [25].

The renin-angiotensin-aldosterone system (RAAS) is one the crucial neurohormonal pathways that plays an important role in the pathophysiology of MetS. After activation of angiotensin-converting enzyme, the adipose tissue produces angiotensin II (Ang II). Increased plasma Ang II levels have been associated with insulin resistance and obesity [26]. In addition, nicotinamide adenine dinucleotide phosphate (NADPH) oxidase is activated by Ang II, which leads to pathogenic consequences that result in an increase in reactive oxygen species (ROS) [27]. LDL oxidation, platelet aggregation, endothelial injury, increased protein levels of nuclear factor kappa-light-chain-enhancer of activated B cells (NF-kB), and synthesis of lipoprotein receptor-1 (LOX-1) on endothelium and vascular smooth muscle cells (VSMCs) are just a few effects of ROS production [28]. RAAS, ROS and LOX-1 have positive feedback that starts a cascade of inflammation, endothelial damage and fibroblast proliferation, which all lead to the progression of insulin resistance, hypertension, dyslipidaemia and CVD (Figure 1).

## 3. Diabetic Retinopathy

Diabetic retinopathy (DR) is one of the most common complications of T2D and is the leading cause of blindness across the globe, resulting in a severe handicap that compromises freedom and has a considerable negative impact on quality of life [29,30]. The multiple risk factors associated with DR include hyperglycaemia, hypertension, dyslipidaemia, pregnancy, puberty, cataract surgery, the progression of diabetes, nephropathy and sleep apnoea syndrome [31]. Based on the degree of microvascular degeneration and associated ischaemic damage, DR can be divided into two forms: non-proliferative DR and advanced, proliferative DR. Diabetic maculopathy is the term used to describe DR that affects the macula, and this form is most common in the latter stages of the disease [32]. Diabetic maculopathy causes vascular leakage and ultimately can lead to vision loss in diabetic patients, which can happen at any stage of the disease [33].

**Figure 2:**
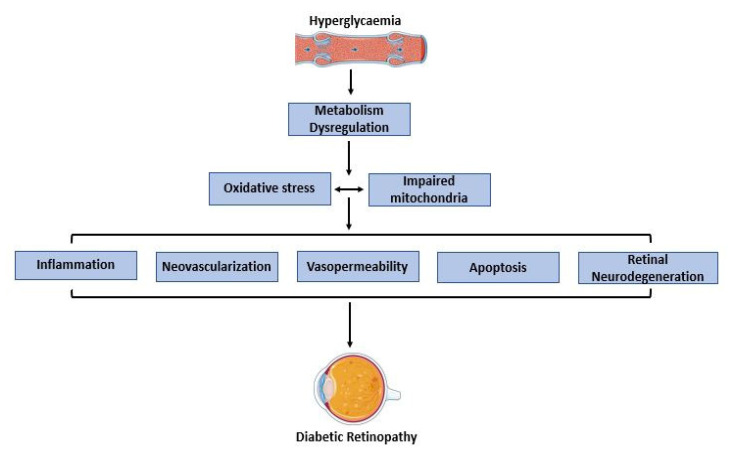
Overview of underlying mechanisms in diabetic retinopathy.

The development of DR is associated with changes in the retinal vasculature, such as progressive damage and subsequent loss of vascular endothelial cells and pericytes, occlusion of capillaries, damage of the blood–retinal barrier, vascular basement membrane thickness, as well as anomalies in neuroretina cells [34]. The key common factor in the pathophysiology of DR is caused by hyperglycaemia, including changes in the calibre and vessel diameter, which result in neovascularization [32]. Retinal neovascularisation occurs as DR progresses. Furthermore, oxidative stress plays a critical role in the development of DR (Figure 2). The retinal arteries may become damaged due to an excessive build-up of circulating ROS, thereby damaging cells of target organs, such as the retina, resulting in DR [35]. It has been shown that four molecular pathways are involved in the pathogenesis of DR: the protein kinase C (PKC) pathway, the hexosamine pathway, the polyol pathway, and accumulation of advanced glycation end products (AGEs). In addition to the aforementioned pathways, epigenetic modifications, dysfunction in the activity of nuclear factors and antioxidant responses, as well as impaired mitochondrial function have been reported with the excessive accumulation of ROS in DR [36,37,38,39]. These pathways not only further increase oxidative stress, but also inflammation, cell death and vascular occlusion, causing upregulation of endothelial growth factors that might contribute to the progression of DR [38].

DR is a complex disease where, in addition to microvascular changes, neurodegeneration plays an important role in the progression of the disease, including apoptosis and glial alteration [40]. Glial activation, decreased retinal neuron function and increased neural apoptosis are all consequences of diabetes-induced neuroglial degeneration that can be seen in diabetic animal models and in post-mortem retina of diabetic patients [41,42]. Alzheimer’s and Parkinson’s diseases share certain overlapping mechanisms with DR, such as glial activation [43]. Müller cells and astrocytes are crucial for regulating retinal homeostasis by controlling the flow of blood through the retina, water balance in the brain parenchyma and vascular permeability [44]. Gliosis often causes blood–retinal barrier malfunction and increased expression of vascular endothelial growth factor (VEGF) and proinflammatory cytokines. As a result, reactive gliosis may be crucial in retinal brain injury and may be associated with neurodegenerative complications [45,46].

## 4. Gut Microbiota

A wide range of microorganisms live in the complex heterogeneous ecosystem known as the human gut microbiome [47]. More than 100 trillion microbial cells make up the intestinal microbial community that resides in the human gut. This community has a mutualistic relationship with its host and plays an important role in the host’s metabolism, for example, producing vitamins and other metabolites required for the host’s physiology [48,49]. Energy, glucose and lipid homeostasis are just a few of the host’s metabolic processes that are regulated by gut microbiota (GM). Numerous studies have shown a causal connection between microbial function and metabolic perturbations and microbial impairment, also known as dysbiosis [50]. Moreover, the variety and abundance of GM play an important role in the composition of intestinal flora [51]. Therefore, decompensation of the intestinal bacteria may result in a variety of diseases, including obesity, inflammatory bowel disorder, asthma, diabetes, neurodegenerative disease, depression and CVD [52,53,54]. According to recent data, changes in the GM work as a critical internal environmental modulator that influences pathogenic mechanisms underlying consequences of T2D, such as DR [55].

T2D is commonly associated with MetS and altered GM, influenced by genetic and environmental factors, ultimately leading to CVD, kidney failure and retinopathy [56]. GM are expected to play an important role in obesity. The composition of GM of obese individuals with and without T2D was significantly different from that of the healthy control group [57]. Changes in *Akkermansia*, *Fecalibacterium*, *Oscillibacter* and *Alistipes*, as well as in serum metabolites, were also related to GM functional capacity and composition [58]. In another study, isolated *Enterobacter clocacae B29* from obese volunteers transplanted to germ-free C57BL/6J mice induced obesity and insulin resistance on a high-fat diet, but not on a regular diet, whereas the germ-free control mice on a high-fat diet did not exhibit the same disease phenotype [59]. These findings demonstrate that GM are crucial regulators of the host’s ability to store fat, which in turn influences the development of obesity. Hyperglycaemia is strongly associated with GM dysbiosis [59]. A microbiome analysis in male Zucker diabetic fatty rats revealed that the progression of the disease and age is linked to changes in faecal microbes. *Firmicutes*, *Bacteroidetes*, *Actinomcrobiota*, and *Proteobacteria* were among the phyla that made up many of the faecal microorganisms in rats at all developmental stages from 8 to 15 weeks. However, in 8- to 10-week-old rats, *Lactobacillus* and *Turicibacter* were the two most common genera [60]. Moderate dysbiosis has been shown in both humans and animals with T2D. Particularly, in T2D individuals, the abundance of some microbiota that are metabolically advantageous, such as butyrate-producing bacteria, is reduced, while the abundance of pathogenic bacteria, which are known to cause several disorders, is severely increased [61]. It has been reported that administration of one specific microbe, i.e., *Akkermansia muciniphila*, a mucin-degrading bacterium that allocates in the mucus layer of gut epithelium, prevents MetS in mice [62,63].

The term dyslipidaemia describes abnormally high levels of lipids or lipoproteins in the blood that can be brought on by inherited or acquired conditions. In vitro and in vivo studies have shown that dyslipidaemia can result in unbalanced GM, and that GM dysbiosis can accelerate lipid metabolic disorders [64]. Mice fed a high-glucose and high-fructose diet showed less diverse GM. The number of Bacteroidetes was low and the fraction of Proteobacteria was noticeably higher in the high-glucose and high-fructose diet groups. Lipid accumulation was greatly increased as well [65]. Rats that developed spontaneous hypertension had considerably lower abundance, variety and homogeneity of GM, but the ratio of Firmicutes/Bacteroidetes increased. A decrease in the number of the microorganisms that generate acetic acid and butyrate coincides with these changes [65].

Purine metabolic abnormalities and reduced uric acid excretion are the two main causes of hyperuricemia. Hyperuricemia is the most significant biochemical cause of gout and MetS symptoms [66]. Studies in humans and experimental animals have revealed a connection between intestinal dysbacteriosis and hyperuricemia [64]. It has been reported that there was a much lower frequency of *Firmicutes* and *Bacteroides* at the phylum level in a mouse model of hyperuricemia. *Bacteroidales*, *Bacteroidaceae*, *Prevotellaceae* and *Rikenellaceae* were more prevalent in this mouse model at the family level. At the genus level, some specific bacterial populations, such as *Clostridium*, *Ruminococcaceae* and *Lactobacillus*, were frequent in the hyperuricemic mice [67]. It has been documented that the GM of gout patients showed increased abundance of the genus *Bacteriodes* and decreased abundance of the genera *Faecalibacterium* and *Bifidobacterium* [68].

The effects of intermittent fasting on the progression of DR, including abnormal growth of capillaries and immune infiltration, have been demonstrated in a study using a diabetic db/db mouse model of diabetes. The intermittent fasting diet augmented levels of Firmicutes; however, both levels of *Verrucomicrobiota* and *Bacteroidetes* were decreased [69]. It has been reported that patients with T2D and DR have altered GM in terms of abundance and diversity. DR patients show lower levels of anti-inflammatory, probiotic, and pathogenic bacteria than healthy individuals and T2D patients [70]. In particular, anti-inflammatory GM, i.e., *Lachnospira*, *Roseburia*, *Coprococcus*, *Phascolarctobacteium*, *Blautia* and *Anerostipes*, were decreased in T2D patients. In the DR group, in addition to the genera, such as *Roseburia*, *Lachnospira* and *Blautia*, several other anti-inflammatory genera, such as *Faecalibacterium*, *Bifidobacterium*, *Ruminococcus*, *Mitsuokella*, *Streptocoocus*, *Lactobacillus* and *Butyrivibrio*, were also decreased. Lipopolysaccharide (LPS) is one of the crucial components of bacterial translocation in chronic inflammation [71]. LPS from *Desulfobacterota* and *Bacteroidetes* has the potential to cause inflammatory damage, leading to impaired metabolism and endotoxin tolerance reduction, all of which are connected to the progression of DR. Moreover, the presence of high levels of *Escherichia coli* (*E.coli*) in the microbiome of T2D patients raises the level of uric acid, which causes the production of ROS, which in turn leads to an extensive damage to retinal endothelial cells and neuronal cell apoptosis, and accelerates the progression of DR [72]. Malfunction of the intestinal flora composition and overproduction of *Heliobacter pylori* induces the expression of both IL-6 and TNF-α, leading to vascular endothelial cell damage. Vascular endothelial cell death causes changes in vascular tone and wall, which activates the complement system and increases vascular permeability, ultimately causing microvascular occlusion and growth of new blood vessels and therefore DR [73].

Recently, a cross-sectional study carried out in Japanese individuals has shown that the composition and functional profiles for GM in T2D patients were significantly different from those in the healthy group [74]. Moreover, the habitual dietary intake, especially sucrose intake, was more strongly associated with these differences, suggesting that reducing sucrose intake might help prevent the onset of T2D through prevention of gut dysbiosis [75]. In the same study, the abundance of *Actinobacteria* was higher in T2D individuals than in the healthy group, while the abundance of *Bacteroidetes* was higher in the healthy group than in T2D individuals. Furthermore, the abundance of *Bacteroides* was lower in T2D patients than in control individuals, whereas the abundance of *Bifidobacterium* was higher in T2D individuals than in the healthy group. Sucrose intake was negatively associated with *Parabacteroides* and *Bacteroides* and positively associated with *Bifidobacterium* [75].

At the genus level, *Roseburia*, *Faecalibacterium*, *Lachnospira* and *Romboustia* were depleted in T2D-DR patients compared with the healthy group, whereas *Akkermansia* was enriched in the T2D-DR group [76]. Furthermore, compared with T2D patients but without DR, the T2D-DR group showed elevated *Prevotella*, *Faecalibacterium*, *Subdoligranulum*, *Agathobacteria* and *Olsenella*, and reduced Bacillus *Veillonella* and *Pantoea* abundances at the genus level. *Faecalibacterium* and *Lachnospira* were depleted in the T2D without DR individuals compared with the healthy group at the genus level, and *Klebsiella* and *Enterococcus* were enriched [76]. Lately, it has been reported at the phylum level, *Actinobacteria* decreased, but *Bacteroidetes* were abundant in T2D-DR patients when compared with those in the healthy group. In addition, at the genus level, *Bifidobacterium* and *Lactobacillus* decreased in faecal samples. Cellular, environmental and metabolism-related pathways were found to be at higher levels in the GM in T2D-DR patients [74].

## 5. Effects of Biological Sex

In various diseases, including CVD, there are fundamental differences between the sexes in manifestation and outcome, as well as the response to treatment [77,78,79,80,81,82,83,84,85,86,87,88,89,90]. Overall, sex differences are generally attributed to genetic and epigenetic mechanisms, regulating major biological processes, including cell death, extracellular matrix deposition and inflammation, as well as sex hormones and their receptors [77,82,83,86,87,88,89,91,92,93,94,95,96,97,98,99,100,101,102,103,104,105,106,107,108,109,110,111,112,113,114,115,116,117,118,119,120,121,122,123,124,125,126,127,128,129]. Along this line, there are significant sex differences in the context of obesity. It is generally thought that women are slightly more likely than men to be obese [130]. However, in contrast to men, women are shielded from various metabolic disorders and sequelae linked to the development of obesity-related disease [131,132]. The patterns of fat deposition, use of fat, and the consequences of both excess and insufficient fat storage vary between men and women. Compared with men, women experience fewer disorders linked to obesity [133]. Sex steroids are important regulators of the relationship between obesity and disease [134,135,136]. In this context, women are less likely to develop T2D before menopause, as they are resistant to free fatty acid-induced insulin release, but this tendency sharply reverses after menopause. Impairment in glucose tolerance, which reflects postprandial insulin resistance, is more common in women than in men, whereas impairment in fasting blood glucose, which reflects fasting insulin resistance, is more common in men than in women [137,138,139].

The difference in the prevalence of diabetes mellitus is related to sex-biased differences in the prevalence of DR. Although type 1 diabetes affects more men than women, no current study has discovered a discernible difference in the prevalence of DR [140]. However, women with DR are more expected to have the proliferative form of diabetes mellitus, and the female sex is an independent risk factor for the development of DR, according to a retrospective analysis of T2D patients under treatment in Japanese clinics [141]. The shift in blood hormone levels during pregnancy is one of the factors to both new cases of retinopathy and the worsening of pre-existing cases [142]. It is believed that oestrogen protects against eye disorders on a preventative basis, most likely as a result of its vasodilatory effects that lower vascular resistance [143]. Nevertheless, it may not always have a preventative effect; unfavourable occurrences like thrombosis might also occur when oestrogen is used as hormonal contraception [144]. In a large clinical study in Denmark, where patients with T2D were followed for several years, it was found that the risk for reaching sight-threatening DR was significantly greater in men [145]. Additionally, men had higher retinal haemorrhages at the baseline examination, higher haemoglobin A1c (HbA1c) levels and higher levels of both diastolic and systolic blood pressure values than women. Since these are known risk factors for developing DR, it may explain why men more frequently develop this disorder [146].

Gonadal hormones have a sex-biased impact on the pathogenesis of T2D. Hormone therapy prevents T2D in menopausal women [131]. Similarly to women, men with low levels of testosterone can delay the onset of T2D through hormone therapy [147]. The maintenance of body composition and metabolism depends heavily on the proper balance of androgens and oestrogens. It is well-established that both androgens and oestrogens play an important role in the bidirectional modulation of glucose metabolism in both sexes. Higher androgen levels in cisgender women are associated with an increase in body weight and visceral fat; female-to-male transgender individuals may experience the same consequences. In general, the incidence of T2D is correlated with considerably greater testosterone levels in women and lower levels in men [147]. Polycystic ovarian syndrome (PCOS) is a condition where women are more likely to experience excess androgen and hyperinsulinemia associated with T2D, obesity and increased CVD risk. The increased prevalence of metabolic disorders in both men and women supports the impact of genetic factors. Obesity is a crucial mediator of impaired glucose metabolism in men and first-degree relatives of women with PCOS [148,149].

Along this line, the composition of GM varies according to sex. In a large cohort from four European countries, men were found to have higher amounts of bacteria from the genera *Prevotella* and *Bacteriodes* than women, which may reflect nutrition patterns and have an impact on weight loss [150,151]. Numerous studies have examined the effects of GM by changing factors like nutrition, medications and lifestyle [152,153,154]. Bacteria from the genera *Slackia* and *Butyricimonas* were significantly linked with oestradiol levels in women, while those from the genera *Acinetobacter*, *Megamonas* and *Ruminococcus* were strongly associated with testosterone levels in men [155]. In addition to exhibiting increased levels of gut inflammation (a typical symptom of obesity and cardiometabolic disease), female mice receiving male donor GM experienced raised levels of testosterone [156,157]. The relationship between sex hormones and GM has been further investigated in animal models using hormones and gonadectomy [158]. Oestradiol is used in hormone therapies to counteract the loss of ovarian oestrogen that menopausal women commonly experience. High-fat diet-fed female mice treated with oestradiol showed lower body mass, improved glucose tolerance and insulin sensitivity as signs of protection from cardiometabolic disorders compared with the corresponding untreated controls. Furthermore, oestradiol modified the GM by reducing the Firmicutes/Bacteroidetes ratio that typically rises in mice on a high-fat diet [158,159]. In addition, male mice treated with oestradiol are less susceptible to gut epithelial permeability, weight gain and inflammation than untreated male mice [160].

## 6. Biomarkers

Biomarkers discovered in blood or other biological fluids and tissues have the power to indicate the presence of illness or an abnormal condition, thereby identifying those who are more likely to develop a disease, those at early stages of disease, as well as monitoring the impact of therapy [161,162]. Sex differences are noticeable in the presence of various fat-related biomarkers. The regulation of satiety, food intake and energy storage depends on leptin. Additionally, leptin affects both peripheral insulin resistance and the insulin glucose pathways [163,164]. Adiponectin influences lipid and glucose metabolism in a variety of ways and improves target organ insulin sensitivity. The progression of T2D is associated with the dysregulation of the function of adiponectin [165]. It has been shown that women with higher adiponectin levels and its receptor in their abdominal adipose tissue might be correlated to a lower CVD risk [166]. Metanalyses have often found that women show higher adiponectin and leptin levels than men of similar body mass index (BMI) and age, which may be associated with sex hormone levels [167]. Increased plasma leptin, which reflects body mass fat and is closely correlated with subcutaneous fat, has been linked in several long-term studies to an increased risk of diabetes in men. However, in obese and diabetic individuals, there is an adverse relationship between insulin sensitivity and plasma adiponectin levels, which is generally more obvious in women [164,166,168]. It has been reported that the hepatokine fetuin A, which was revealed to be associated with T2D onset solely in women, is one of the novel risk factors related to sex-related differences [169].

Copeptin has been shown to be associated with the risk of developing T2D in both men and women [170]. This biomarker is the C-terminal component of the precursor of vasopressin and a reliable marker of arginine vasopressin secretion [169]. This may indicate a closer connection between the pathophysiology of T2D and the arginine vasopressin stress adaptation system. After consuming fat, the small intestine’s endocrine-like N-cells peripherally release proneurotensin, which functions as a neurotransmitter in the central nervous system. However, in the peripheral neurological system, it works as a hormone, boosting pancreatic and biliary secretion, decreasing gastric motility, and facilitating fatty acid translocation. Fasting plasma levels of proneurotensin are typically lower in women than in men, but they predict incident CVD and diabetes, as well as overall CVD mortality in women but not in men [171,172]. Low 25(OH) vitamin D3 was shown in middle-aged Caucasians to be linked with T2D in women but not in men. Women with 25(OH) vitamin D3 levels below the cut-off of 15 ng/mL doubled their risk of having recently diagnosed diabetes [173].

Based on their involvement in the progression of DR, biomarkers for DR in the systemic circulation or local tissues may serve as an indicator of pathological processes related to DR [174]. They can now be measured in tears in addition to blood, lens, cornea, retina, vitreous and aqueous humor [175]. There is a big number of serum proteins that have been found to be altered in DR, such as IL-6, TNF-α, pigment epithelium-derived factor (PEDF), plasminogen activator inhibitor 1 (PAI-1), vascular adhesion endothelial molecule 1 (VCAM-1) and VEGF [176,177,178]. VEGF is one of the most well-known biomarkers associated with retinopathies [177]. Its expression causes retinal injury and results in choroidal neovascularization [179]. CRP, VCAM-1, intercellular adhesion molecule 1 (ICAM-1), soluble glycoprotein 130 (sgp 130) and TNF receptor 1 have been found to be significantly higher in the serum of T1D individuals with DR compared with those without DR [180]. The vitreous humor contains several proteins that have been identified as DR biomarkers at various stages of the disease. Numerous studies have found components of the acute phase response, coagulation pathway, complement system and other inflammatory pathways in DR patients [181,182].

Antimicrobial peptides, inflammatory peptides and short-chain fatty acids (SCFA) levels are examples of faecal biomarkers that are emerging as non-invasive screening tools for determining and diagnosing medical disorders [183]. Understanding the relationship between the host and GM has been enriched thanks to metabolomics, which involves the analysis of small molecules found in any type of biological sample. Several clinical characteristics are related with metabolic diseases with hundreds of serum or faecal metabolites [184]. It has been shown that a decrease in the synthesis of *Bacteroides thetaiotaomicron*, a glutamate fermenting commensal, in individuals with obesity is inversely associated with serum glutamate [185]. Moreover, positive associations between microbial function and insulin resistance are driven by *Prevotella copri* and *Bacteriodes vulgatus*, among others, indicating that they may play a role in host metabolism [185]. A comprehensive analysis of the oral microbiome revealed *Granulicatella* and Neisseria as bacteria enriched in MetS patients and *Peptococcus* as bacteria abundant in the healthy group [186].

## 7. Sex-Biased Effects of Gut Microbiota in Metabolic Syndrome-Related Diabetic Retinopathy

*Actinobacteria*, *Firmicutes*, *Bacteroidetes*, *Proteobacteria*, *Fusobacteria* and *Verrucomicrobia* are the six phyla that make up most of the bacteria in the gut, with Bacteroidetes and Firmicutes accounting for 90% of the total GM composition [187]. Numerous conditions are frequently linked to variations in the prevalence of Firmicutes and Bacteroidetes, as well as the overall increase or decrease in the Firmicutes:Bacteriodetes ratio [188]. It has been reported that a decrease in the abundance of *A. muciniphila* increases gut permeability, a known feature in T2D [189]. The decrease in this group is consistent with a study comparing the GM patterns of women with active versus sedentary lifestyles, which found an increase in this genus, as well as other species of bacteria that are health-promoting, including *Roseburia hominis*, *Faecalibacterium prausnitzii* and *Akkermansia muciniphila* in women with an active lifestyle [190]. Vascular disorders are a leading cause of morbidity and mortality in both men and women with T1D and T2D. However, the incidence, development and pathophysiology of both microvascular and macrovascular complications differ between the sexes. Importantly, the presence of diabetes confers higher risk for vascular consequences in women compared with men, and the contribution of sex hormones and sex-biased risk factors play an important role [191]. A distinctive GM profile in T2D associated with DR individuals has been identified [76].

Interestingly, it has been shown in both humans and mice that mitochondrial adipose tissue functions are increased in females and closely associated with adiposity, insulin resistance and plasma lipids. A genetic locus on mouse chromosome 17 was identified that regulates mitochondrial mass and function in adipose tissue in a sex- and tissue-specific manner by using a panel of diverse inbred strains of mice [192]. This locus contains the *Ndufv2* (NADH: ubiquinone oxidoreductase core subunit 2) gene, which regulates the expression of at least 89 mitochondrial genes in females, including oxidative phosphorylation genes and those related to mitochondrial DNA content [192]. In the hybrid mouse diversity panel (HMDP), which consists of a panel of about 100 genetically diverse inbred mouse strains [193], female HMDP mice were found to have higher adipose expression of oxidative phosphorylation (OXPHOS) genes compared with males. In addition, similar tissue-by-sex interactions in human samples were observed, with the subcutaneous adipose tissue of women showing higher expression of OXPHOS genes compared with that of men. Moreover, mitochondrial DNA (mtDNA) levels were strongly associated with metabolic traits, for instance the homeostatic model for insulin resistance (HOMA-IR), in females [192]. To summarize, the regulation of adipose mitochondrial function in a tissue- and sex-specific manner has a significant impact on several metabolic variables linked to T2D and CVD in humans and mice. The complex I protein NDUFV2 was also identified as an important contributor to sex differences. Another study has showed increased levels of the brown adipocyte marker uncoupling protein 1 in women, which indicates that the higher relative contribution of the fat mass to the resting metabolic rate (RMR) in women may be explained by an increased number of brown adipocytes shown in this group [194].

## 8. Conclusions

Metabolic disorders are strongly associated with impaired GM in a sex-dependent manner. Studies have shown that in both animal models and humans with diabetes and its consequences, such as DR, there are different GM compositions compared with controls, affected by biological sex. Macrovascular and microvascular disorders might result from uncontrolled diabetes, as well as metabolic problems associated with lipid metabolism dysregulation, hypertension and excessive ROS production. One of the most common microvascular consequences of diabetes is DR, which can ultimately lead to blindness. Currently, there are a few therapeutic options to treat DR: laser photocoagulation, intravitreal corticosteroids and intravitreal anti-VEGF. However, these treatments are mainly focused on later stage disease [195], and the effects of biological sex are poorly understood.

Both prevention and treatment of MetS involve lifestyle changes and therapeutic agents together to decrease CVD risk. In addition to medications lowering insulin demand and enhancing insulin responsiveness [4], nutritional interventions, including calorie restriction, are also important in the treatment of insulin resistance. An improvement in glucose metabolism, insulin sensitivity and suppression of pro-inflammatory cytokines have been associated with *Lactobacillus fermentum*, *plantarum* and *casei*, *Roseburia intestinalis*, *Akkermansia muciniphila* and *Bacteriodes fragilis* [196]. Metformin, which is frequently used to treat diabetes, has been shown to modulate the composition of the GM [197]. In preclinical models of DR, altering the GM by administering probiotics has demonstrated beneficial effects. It has been shown that administration of recombinant *Lactobacillus paracasei* in mice with DR decreased cell loss and inflammatory cytokine production in the retina [198]. In parallel, administration of *Lactobacillus paracasei* secreting Ang1-7 in diabetic mice improved glucose tolerance and reduced diabetes-induced damage in the kidney and retina [199]. Controlling GM through probiotics, prebiotics, synbiotics, or faecal microbiota could be a successful way of treating diabetes and its consequences. However, further research and clinical trials, including appropriate representation of female individuals, are necessary. Identifying bacterial signatures and metabolites will enable early detection of disease risks and mechanisms, allowing for personalisation of healthcare interventions based on individual stage, needs and complications of disease in both men and women.

## Data Availability

Not applicable.

## References

[1] Huang P.L. (2009). A comprehensive definition for metabolic syndrome. Dis. Model. Mech..

[2] Rochlani Y., Pothineni N.V., Kovelamudi S., Mehta J.L. (2017). Metabolic syndrome: Pathophysiology, management, and modulation by natural compounds. Ther. Adv. Cardiovasc. Dis..

[3] Saely C.H., Aczel S., Marte T., Langer P., Hoefle G., Drexel H. (2005). The metabolic syndrome, insulin resistance, and cardiovascular risk in diabetic and nondiabetic patients. J. Clin. Endocrinol. Metab..

[4] Fahed G., Aoun L., Bou Zerdan M., Allam S., Bou Zerdan M., Bouferraa Y., Assi H.I. (2022). Metabolic syndrome: Updates on pathophysiology and management in 2021. Int. J. Mol. Sci..

[5] Lemieux I., Després J.-P. (2020). Metabolic syndrome: Past, present and future. Nutrients.

[6] Wang H.H., Lee D.K., Liu M., Portincasa P., Wang D.Q.-H. (2020). Novel Insights into the Pathogenesis and Management of the Metabolic Syndrome. Pediatr. Gastroenterol. Hepatol. Nutr..

[7] Beltrán-Sánchez H., Harhay M.O., Harhay M.M., McElligott S. (2013). Prevalence and trends of metabolic syndrome in the adult U.S. population, 1999–2010. J. Am. Coll. Cardiol..

[8] Khambaty T., Schneiderman N., Llabre M.M., Elfassy T., Moncrieft A.E., Daviglus M., Talavera G.A., Isasi C.R., Gallo L.C., Reina S.A. (2020). Elucidating the multidimensionality of socioeconomic status in relation to metabolic syndrome in the hispanic community health study/study of latinos (HCHS/SOL). Int. J. Behav. Med..

[9] Santos A.C., Ebrahim S., Barros H. (2008). Gender, socio-economic status and metabolic syndrome in middle-aged and old adults. BMC Public Health.

[10] Yang Y.C., Schorpp K., Boen C., Johnson M., Harris K.M. (2018). Socioeconomic status and biological risks for health and illness across the life course. J. Gerontol. Ser. B.

[11] Abbate M., Pericas J., Yañez A.M., López-González A.A., De Pedro-Gómez J., Aguilo A., Morales-Asencio J.M., Bennasar-Veny M. (2021). Socioeconomic inequalities in metabolic syndrome by age and gender in a spanish working population. Int. J. Environ. Res. Public Health.

[12] Krieger N. (2003). Genders, sexes, and health: What are the connections—And why does it matter?. Leuk. Res..

[13] Kahn C.R., Wang G., Lee K.Y. (2019). Altered adipose tissue and adipocyte function in the pathogenesis of metabolic syndrome. J. Clin. Investig..

[14] Mauvais-Jarvis F., Manson J.E., Stevenson J.C., Fonseca V.A. (2017). Menopausal hormone therapy and type 2 diabetes prevention: Evidence, mechanisms, and clinical implications. Endocr. Rev..

[15] Deacon C.F. (2019). Physiology and pharmacology of dpp-4 in glucose homeostasis and the treatment of type 2 diabetes. Front. Endocrinol..

[16] Cade W.T. (2008). Diabetes-related microvascular and macrovascular diseases in the physical therapy setting. Phys. Ther..

[17] Chawla R., Chawla A., Jaggi S. (2016). Microvasular and macrovascular complications in diabetes mellitus: Distinct or continuum?. Indian J. Endocrinol. Metab..

[18] Kyriazis I.D., Hoffman M., Gaignebet L., Lucchese A.M., Markopoulou E., Palioura D., Wang C., Bannister T.D., Christofidou-Solomidou M., Oka S.-I. (2021). KLF5 is induced by FOXO1 and causes oxidative stress and diabetic cardiomyopathy. Circ. Res..

[19] Lima V.C., Cavalieri G.C., Lima M.C., Nazario N.O., Lima G.C. (2016). Risk factors for diabetic retinopathy: A case–control study. Int. J. Retin. Vitr..

[20] Scheithauer T.P.M., Rampanelli E., Nieuwdorp M., Vallance B.A., Verchere C.B., van Raalte D.H., Herrema H. (2020). Gut microbiota as a trigger for metabolic inflammation in obesity and type 2 diabetes. Front. Immunol..

[21] Li S., Kararigas G. (2022). Role of biological sex in the cardiovascular-gut microbiome axis. Front. Cardiovasc. Med..

[22] Agnoletti D., Piani F., Cicero A.F.G., Borghi C. (2022). The Gut Microbiota and Vascular Aging: A State-of-the-Art and Systematic Review of the Literature. J. Clin. Med..

[23] Ruan H., Lodish H.F. (2003). Insulin resistance in adipose tissue: Direct and indirect effects of tumor necrosis factor-α. Cytokine Growth Factor Rev..

[24] Tsigos C., Kyrou I., Chala E., Tsapogas P., Stavridis J.C., Raptis S.A., Katsilambros N. (1999). Circulating tumor necrosis factor alpha concentrations are higher in abdominal versus peripheral obesity. Metabolism.

[25] Bastard J.-P., Hainque B. (2000). Relationship between plasma plasminogen activator inhibitor 1 and insulin resistance. Diabetes/Metab. Res. Rev..

[26] Rajagopalan S., Kurz S., Münzel T., Tarpey M., Freeman B.A., Griendling K.K., Harrison D.G. (1996). Angiotensin II-mediated hypertension in the rat increases vascular superoxide production via membrane NADH/NADPH oxidase activation. Contribution to alterations of vasomotor tone. J. Clin. Investig..

[27] Huang S.-S., Lu Y.-J., Huang J.-P., Wu Y.-T., Day Y.-J., Hung L.-M. (2014). The essential role of endothelial nitric oxide synthase activation in insulin-mediated neuroprotection against ischemic stroke in diabetes. J. Vasc. Surg..

[28] Li D.Y., Zhang Y.C., Philips M.I., Sawamura T., Mehta J.L. (1999). Upregulation of endothelial receptor for oxidized low-density lipoprotein (LOX-1) in cultured human coronary artery endothelial cells by angiotensin II type 1 receptor activation. Circ. Res..

[29] Ting D.S.W., Cheung C.M.G., Wong T.Y. (2016). Diabetic retinopathy: Global prevalence, major risk factors, screening practices and public health challenges: A review. Clin. Exp. Ophthalmol..

[30] Zheng Y., He M., Congdon N. (2012). The worldwide epidemic of diabetic retinopathy. Indian J. Ophthalmol..

[31] Jenkins A.J., Joglekar M.V., Hardikar A.A., Keech A.C., O’Neal D.N., Januszewski A.S. (2015). Biomarkers in diabetic retinopathy. Rev. Diabet. Stud..

[32] Lechner J., O’Leary O.E., Stitt A.W. (2017). The pathology associated with diabetic retinopathy. Vis. Res..

[33] Stitt A.W., Curtis T.M., Chen M., Medina R.J., McKay G.J., Jenkins A., Gardiner T.A., Lyons T.J., Hammes H.-P., Simó R. (2016). The progress in understanding and treatment of diabetic retinopathy. Prog. Retin. Eye Res..

[34] Simó R., Stitt A.W., Gardner T.W. (2018). Neurodegeneration in diabetic retinopathy: Does it really matter?. Diabetologia.

[35] Kang Q., Yang C. (2020). Oxidative stress and diabetic retinopathy: Molecular mechanisms, pathogenetic role and therapeutic implications. Redox Biol..

[36] Miller W.P., Sunilkumar S., Giordano J.F., Toro A.L., Barber A.J., Dennis M.D. (2020). The stress response protein REDD1 promotes diabetes-induced oxidative stress in the retina by Keap1-independent Nrf2 degradation. J. Biol. Chem..

[37] Sabharwal S.S., Schumacker P.T. (2014). Mitochondrial ROS in cancer: Initiators, amplifiers or an Achilles’ heel?. Nat. Rev. Cancer.

[38] Safi S.Z., Qvist R., Kumar S., Batumalaie K., Ismail I.S.B. (2014). Molecular mechanisms of diabetic retinopathy, general preventive strategies, and novel therapeutic targets. Bio. Med. Res. Int..

[39] Sui A., Chen X., Demetriades A.M., Shen J., Cai Y., Yao Y., Yao Y., Zhu Y., Shen X., Xie B. (2020). Inhibiting NF-κB signaling activation reduces retinal neovascularization by promoting a polarization shift in macrophages. Investig. Opthalmology Vis. Sci..

[40] Madeira M.H., Marques I.P., Ferreira S., Tavares D., Santos T., Santos A.R., Figueira J., Lobo C., Cunha-Vaz J. (2021). Retinal neurodegeneration in different risk phenotypes of diabetic retinal disease. Front. Neurosci..

[41] Carrasco E., Hernández C., Miralles A., Huguet P., Farrés J., Simó R. (2007). Lower somatostatin expression is an early event in diabetic retinopathy and is associated with retinal neurodegeneration. Diabetes Care.

[42] Lieth E., Barber A.J., Xu B., Dice C., Ratz M.J., Tanase D., Strother J.M. (1998). Glial reactivity and impaired glutamate metabolism in short-term experimental diabetic retinopathy. Penn State Retina Research Group. Diabetes.

[43] Xie A., Gao J., Xu L., Meng D. (2014). Shared mechanisms of neurodegeneration in alzheimer’s disease and parkinson’s disease. Bio. Med. Res. Int..

[44] Sundstrom J.M., Hernández C., Weber S.R., Zhao Y., Dunklebarger M., Tiberti N., Laremore T., Simó-Servat O., Garcia-Ramirez M., Barber A.J. (2018). Proteomic analysis of early diabetic retinopathy reveals mediators of neurodegenerative brain diseases. Investig. Ophthalmol. Vis. Sci..

[45] Bringmann A., Wiedemann P. (2011). Müller glial cells in retinal disease. Ophthalmologica.

[46] Wang L., Liu M., Gao J., Smith A.M., Fujioka H., Liang J., Perry G., Wang X. (2021). Mitochondrial fusion suppresses tau pathology-induced neurodegeneration and cognitive decline. J. Alzheimer’s Dis..

[47] Fan Y., Pedersen O. (2021). Gut microbiota in human metabolic health and disease. Nat. Rev. Microbiol..

[48] Bermon S., Petriz B., Kajėnienė A., Prestes J., Castell L., Franco O.L. (2015). The microbiota: An exercise immunology perspective. Exerc. Immunol. Rev..

[49] McQuade J.L., Daniel C.R., Helmink B.A., Wargo J.A. (2019). Modulating the microbiome to improve therapeutic response in cancer. Lancet Oncol..

[50] Schoeler M., Caesar R. (2019). Dietary lipids, gut microbiota and lipid metabolism. Rev. Endocr. Metab. Disord..

[51] Chen Y., Zhou J., Wang L. (2021). Role and mechanism of gut microbiota in human disease. Front. Cell. Infect. Microbiol..

[52] Kim J.-M., Stewart R., Kim J.-W., Kang H.-J., Bae K.-Y., Kim S.-W., Shin I.-S., Yoon J.-S. (2018). Changes in pro-inflammatory cytokine levels and late-life depression: A two year population based longitudinal study. Psychoneuroendocrinology.

[53] Sherwin E., Dinan T.G., Cryan J.F. (2017). Recent developments in understanding the role of the gut microbiota in brain health and disease. Ann. Acad. Sci..

[54] Xu Y., Zhou H., Zhu Q. (2017). The Impact of microbiota-gut-brain axis on diabetic cognition impairment. Front. Aging Neurosci..

[55] Huang Y., Wang Z., Ma H., Ji S., Chen Z., Cui Z., Chen J., Tang S. (2021). Dysbiosis and implication of the gut microbiota in diabetic retinopathy. Front. Cell. Infect. Microbiol..

[56] Luca M., Di Mauro M., Di Mauro M., Luca A. (2019). Gut Microbiota in alzheimer’s disease, depression, and type 2 diabetes mellitus: The role of oxidative stress. Oxidative Med. Cell. Longev..

[57] Kim M.-H., Yun K.E., Kim J., Park E., Chang Y., Ryu S., Kim H.-L., Kim H.-N. (2020). Gut microbiota and metabolic health among overweight and obese individuals. Sci. Rep..

[58] Thingholm L.B., Rühlemann M.C., Koch M., Fuqua B., Laucke G., Boehm R., Bang C., Franzosa E.A., Hübenthal M., Rahnavard G. (2019). Obese individuals with and without type 2 diabetes show different gut microbial functional capacity and composition. Cell Host Microbe.

[59] Fei N., Zhao L. (2012). An opportunistic pathogen isolated from the gut of an obese human causes obesity in germfree mice. ISME J..

[60] Zhou W., Xu H., Zhan L., Lu X., Zhang L. (2019). Dynamic development of fecal microbiome during the progression of diabetes mellitus in zucker diabetic fatty rats. Front. Microbiol..

[61] Qin J., Li Y., Cai Z., Li S., Zhu J., Zhang F., Liang S., Zhang W., Guan Y., Shen D. (2012). A metagenome-wide association study of gut microbiota in type 2 diabetes. Nature.

[62] Everard A., Cani P.D. (2013). Diabetes, obesity and gut microbiota. Best Pract. Res. Clin. Gastroenterol..

[63] Plovier H., Everard A., Druart C., Depommier C., Van Hul M., Geurts L., Chilloux J., Ottman N., Duparc T., Lichtenstein L. (2016). A purified membrane protein from *Akkermansia muciniphila* or the pasteurized bacterium improves metabolism in obese and diabetic mice. Nat. Med..

[64] Wang P.-X., Deng X.-R., Zhang C.-H., Yuan H.-J. (2020). Gut microbiota and metabolic syndrome. Chin. Med. J..

[65] Do M.H., Lee E., Oh M.-J., Kim Y., Park H.-Y. (2018). High-glucose or-fructose diet cause changes of the gut microbiota and metabolic disorders in mice without body weight change. Nutrients.

[66] Copur S., Demiray A., Kanbay M. (2022). Uric acid in metabolic syndrome: Does uric acid have a definitive role?. Eur. J. Intern. Med..

[67] Xu D., Lv Q., Wang X., Cui X., Zhao P., Yang X., Liu X., Yang W., Yang G., Wang G. (2019). Hyperuricemia is associated with impaired intestinal permeability in mice. Am. J. Physiol. Liver Physiol..

[68] Guo Z., Zhang J., Wang Z., Ang K.Y., Huang S., Hou Q., Su X., Qiao J., Zheng Y., Wang L. (2016). Intestinal microbiota distinguish gout patients from healthy humans. Sci. Rep..

[69] Beli E., Yan Y., Moldovan L., Vieira C.P., Gao R., Duan Y., Prasad R., Bhatwadekar A., White F.A., Townsend S.D. (2018). Restructuring of the gut microbiome by intermittent fasting prevents retinopathy and prolongs survival in db/db mice. Diabetes.

[70] Das T., Jayasudha R., Chakravarthy S., Prashanthi G.S., Bhargava A., Tyagi M., Rani P.K., Pappuru R.R., Sharma S., Shivaji S. (2021). Alterations in the gut bacterial microbiome in people with type 2 diabetes mellitus and diabetic retinopathy. Sci. Rep..

[71] Layoun A., Santos M.M. (2012). Bacterial cell wall constituents induce hepcidin expression in macrophages through myd88 signaling. Inflammation.

[72] Moubayed N.M., Bhat R.S., Al Farraj D., Al Dihani N., El Ansary A., Fahmy R.M. (2019). Screening and identification of gut anaerobes (Bacteroidetes) from human diabetic stool samples with and without retinopathy in comparison to control subjects. Microb. Pathog..

[73] Sayed K.M., Mahmoud A.A. (2016). Heat shock protein-70 and hypoxia inducible factor-1αin type 2 diabetes mellitus patients complicated with retinopathy. Acta Ophthalmol..

[74] Li L., Yang K., Li C., Zhang H., Yu H., Chen K., Yang X., Liu L. (2022). Metagenomic shotgun sequencing and metabolomic profiling identify specific human gut microbiota associated with diabetic retinopathy in patients with type 2 diabetes. Front. Immunol..

[75] Hashimoto Y., Hamaguchi M., Kaji A., Sakai R., Osaka T., Inoue R., Kashiwagi S., Mizushima K., Uchiyama K., Takagi T. (2020). Intake of sucrose affects gut dysbiosis in patients with type 2 diabetes. J. Diabetes Investig..

[76] Zhou Z., Zheng Z., Xiong X., Chen X., Peng J., Yao H., Pu J., Chen Q., Zheng M. (2021). Gut microbiota composition and fecal metabolic profiling in patients with diabetic retinopathy. Front. Cell Dev. Biol..

[77] Gaignebet L., Kararigas G. (2017). En route to precision medicine through the integration of biological sex into pharmacogenomics. Clin. Sci..

[78] Kararigas G., Seeland U., De Arellano M.L.B., Dworatzek E., Regitz-Zagrosek V. (2016). Why the study of the effects of biological sex is important. Ann. Dell’istituto Super. Di Sanità..

[79] Cui C., Huang C., Liu K., Xu G., Yang J., Zhou Y., Feng Y., Kararigas G., Geng B., Cui Q. (2018). Large-scale in silico identification of drugs exerting sex-specific effects in the heart. J. Transl. Med..

[80] Ruiz-Meana M., Boengler K., Garcia-Dorado D., Hausenloy D.J., Kaambre T., Kararigas G., Perrino C., Schulz R., Ytrehus K. (2019). Ageing, sex, and cardioprotection. Br. J. Pharmacol..

[81] Altinbas L., Bormann N., Lehmann D., Jeuthe S., Wulsten D., Kornak U., Robinson P.N., Wildemann B., Kararigas G. (2019). Assessment of bones deficient in fibrillin-1 microfibrils reveals pronounced sex differences. Int. J. Mol. Sci..

[82] Regitz-Zagrosek V., Kararigas G. (2017). Mechanistic pathways of sex differences in cardiovascular disease. Physiol. Rev..

[83] Ober C., Loisel D.A., Gilad Y. (2008). Sex-specific genetic architecture of human disease. Nat. Rev. Genet..

[84] McCarthy M.M., Nugent B.M., Lenz K.M. (2017). Neuroimmunology and neuroepigenetics in the establishment of sex differences in the brain. Nat. Rev. Neurosci..

[85] Kokras N., Hodes G.E., Bangasser D.A., Dalla C. (2019). Sex differences in the hypothalamic–pituitary–adrenal axis: An obstacle to antidepressant drug development?. Br. J. Pharmacol..

[86] Kararigas G., Dworatzek E., Petrov G., Summer H., Schulze T.M., Baczko I., Knosalla C., Golz S., Hetzer R., Regitz-Zagrosek V. (2014). Sex-dependent regulation of fibrosis and inflammation in human left ventricular remodelling under pressure overload. Eur. J. Heart Fail..

[87] Gaignebet L., Kańduła M.M., Lehmann D., Knosalla C., Kreil D.P., Kararigas G. (2020). Sex-specific human cardiomyocyte gene regulation in left ventricular pressure overload. Mayo Clin. Proc..

[88] Dworatzek E., Baczko I., Kararigas G. (2015). Effects of aging on cardiac extracellular matrix in men and women. Proteom.–Clin. Appl..

[89] Petrov G., Dworatzek E., Schulze T.M., Dandel M., Kararigas G., Mahmoodzadeh S., Knosalla C., Hetzer R., Regitz-Zagrosek V. (2014). Maladaptive remodeling is associated with impaired survival in women but not in men after aortic valve replacement. JACC Cardiovasc. Imaging.

[90] Siokatas G., Papatheodorou I., Daiou A., Lazou A., Hatzistergos K.E., Kararigas G. (2022). Sex-related effects on cardiac development and disease. J. Cardiovasc. Dev. Dis..

[91] Pei J., Harakalova M., Treibel T., Lumbers R.T., Boukens B.J., Efimov I.R., Van Dinter J.T., González A., López B., El Azzouzi H. (2020). H3K27ac acetylome signatures reveal the epigenomic reorganization in remodeled non-failing human hearts. Clin. Epigenetics.

[92] Iorga A., Cunningham C.M., Moazeni S., Ruffenach G., Umar S., Eghbali M. (2017). The protective role of estrogen and estrogen receptors in cardiovascular disease and the controversial use of estrogen therapy. Biol. Sex Differ..

[93] Murphy E. (2011). Estrogen signaling and cardiovascular disease. Circ. Res..

[94] Murphy E., Steenbergen C. (2014). Estrogen regulation of protein expression and signaling pathways in the heart. Biol. Sex Differ..

[95] Menazza S., Murphy E. (2016). The expanding complexity of estrogen receptor signaling in the cardiovascular system. Circ. Res..

[96] Puglisi R., Mattia G., Carè A., Marano G., Malorni W., Matarrese P. (2019). Non-genomic effects of estrogen on cell homeostasis and remodeling with special focus on cardiac ischemia/reperfusion injury. Front. Endocrinol..

[97] Lowe D.A., Kararigas G. (2020). Editorial: New insights into estrogen/estrogen receptor effects in the cardiac and skeletal muscle. Front. Endocrinol..

[98] Kalkhoran S.B., Kararigas G. (2022). Oestrogenic regulation of mitochondrial dynamics. Int. J. Mol. Sci..

[99] Ruijter H.M.D., Kararigas G. (2022). Estrogen and cardiovascular health. Front. Cardiovasc. Med..

[100] Kararigas G. (2021). Oestrogenic contribution to sex-biased left ventricular remodelling: The male implication. Int. J. Cardiol..

[101] Schubert C., Raparelli V., Westphal C., Dworatzek E., Petrov G., Kararigas G., Regitz-Zagrosek V. (2016). Reduction of apoptosis and preservation of mitochondrial integrity under ischemia/reperfusion injury is mediated by estrogen receptor beta. Biol. Sex Differ..

[102] Mahmoodzadeh S., Dworatzek E. (2019). The role of 17β-estradiol and estrogen receptors in regulation of Ca^2+^ channels and mitochondrial function in cardiomyocytes. Front. Endocrinol..

[103] Sickinghe A.A., Korporaal S.J.A., Ruijter H.M.D., Kessler E.L. (2019). Estrogen contributions to microvascular dysfunction evolving to heart failure with preserved ejection fraction. Front. Endocrinol..

[104] Ventura-Clapier R., Piquereau J., Veksler V., Garnier A. (2019). Estrogens, estrogen receptors effects on cardiac and skeletal muscle mitochondria. Front. Endocrinol..

[105] Zhang B., Miller V.M., Miller J. (2019). Influences of sex and estrogen in arterial and valvular calcification. Front. Endocrinol..

[106] Kararigas G., Fliegner D., Forler S., Klein O., Schubert C., Gustafsson J., Klose J., Regitz-Zagrosek V. (2014). Comparative proteomic analysis reveals sex and estrogen receptor β effects in the pressure overloaded heart. J. Proteome Res..

[107] Kararigas G., Nguyen B.T., Jarry H. (2014). Estrogen modulates cardiac growth through an estrogen receptor α-dependent mechanism in healthy ovariectomized mice. Mol. Cell. Endocrinol..

[108] Kararigas G., Nguyen B.T., Zelarayán L.C., Hassenpflug M., Toischer K., Sanchez-Ruderisch H., Hasenfuss G., Bergmann M.W., Jarry H., Regitz-Zagrosek V. (2014). Genetic background defines the regulation of postnatal cardiac growth by 17β-Estradiol through a β-catenin mechanism. Endocrinology.

[109] Kararigas G., Fliegner D., Gustafsson J., Regitz-Zagrosek V. (2011). Role of the estrogen/estrogen-receptor-beta axis in the genomic response to pressure overload-induced hypertrophy. Physiol. Genom..

[110] Sanchez-Ruderisch H., Queirós A.M., Fliegner D., Eschen C., Kararigas G., Regitz-Zagrosek V. (2019). Sex-specific regulation of cardiac microRNAs targeting mitochondrial proteins in pressure overload. Biol. Sex Differ..

[111] Duft K., Schanz M., Pham H., Abdelwahab A., Schriever C., Kararigas G., Dworatzek E., Davidson M.M., Regitz-Zagrosek V., Morano I. (2017). 17beta-Estradiol-induced interaction of estrogen receptor alpha and human atrial essential myosin light chain modulates cardiac contractile function. Basic Res. Cardiol..

[112] Lai S., Collins B.C., Colson B.A., Kararigas G., Lowe D.A. (2016). Estradiol modulates myosin regulatory light chain phosphorylation and contractility in skeletal muscle of female mice. Am. J. Physiol. Metab..

[113] Mahmoodzadeh S., Pham T.H., Kuehne A., Fielitz B., Dworatzek E., Kararigas G., Petrov G., Davidson M.M., Regitz-Zagrosek V. (2012). 17β-Estradiol-induced interaction of ERα with NPPA regulates gene expression in cardiomyocytes. Cardiovasc. Res..

[114] Nguyen B.T., Kararigas G., Jarry H. (2012). Dose-dependent effects of a genistein-enriched diet in the heart of ovariectomized mice. Genes Nutr..

[115] Nguyen B.T., Kararigas G., Wuttke W., Jarry H. (2011). Long-term treatment of ovariectomized mice with estradiol or phytoestrogens as a new model to study the role of estrogenic substances in the heart. Planta Med..

[116] Kararigas G., Bito V., Tinel H., Becher E., Baczko I., Knosalla C., Albrecht-Küpper B., Sipido K.R., Regitz-Zagrosek V. (2012). Transcriptome characterization of estrogen-treated human myocardium identifies myosin regulatory light chain interacting protein as a sex-specific element influencing contractile function. J. Am. Coll. Cardiol..

[117] Kararigas G., Becher E., Mahmoodzadeh S., Knosalla C., Hetzer R., Regitz-Zagrosek V. (2010). Sex-specific modification of progesterone receptor expression by 17β-oestradiol in human cardiac tissues. Biol. Sex Differ..

[118] Hein S., Hassel D., Kararigas G. (2019). The zebrafish (danio rerio) is a relevant model for studying sex-specific effects of 17β-estradiol in the adult heart. Int. J. Mol. Sci..

[119] Fliegner D., Schubert C., Penkalla A., Witt H., Kararigas G., Dworatzek E., Staub E., Martus P., Noppinger P.R., Kintscher U. (2010). Female sex and estrogen receptor-β attenuate cardiac remodeling and apoptosis in pressure overload. Am. J. Physiol. Integr. Comp. Physiol..

[120] Queirós A.M., Eschen C., Fliegner D., Kararigas G., Dworatzek E., Westphal C., Ruderisch H.S., Regitz-Zagrosek V. (2013). Sex-and estrogen-dependent regulation of a miRNA network in the healthy and hypertrophied heart. Int. J. Cardiol..

[121] Kararigas G. (2022). Sex-biased mechanisms of cardiovascular complications in COVID-19. Physiol. Rev..

[122] Ritter O., Kararigas G. (2020). Sex-Biased Vulnerability of the Heart to COVID-19. Mayo Clin. Proc..

[123] Lam C.S., Carson P.E., Anand I., Rector T.S., Kuskowski M., Komajda M., McKelvie R.S., Mcmurray J., Zile M.R., Massie B.M. (2012). Sex differences in clinical characteristics and outcomes in elderly patients with heart failure and preserved ejection fraction: The irbesartan in heart failure with preserved ejection fraction (I-PRESERVE) trial. Circ. Heart Fail..

[124] Beale A.L., Meyer P., Marwick T.H., Lam C.S., Kaye D.M. (2018). Sex differences in cardiovascular pathophysiology: Why women are overrepresented in heart failure with preserved ejection fraction. Circulation.

[125] Sabbatini A.R., Kararigas G. (2020). Menopause-related estrogen decrease and the pathogenesis of HFpEF: JACC review topic of the week. J. Am. Coll. Cardiol..

[126] Sabbatini A.R., Kararigas G. (2020). Estrogen-related mechanisms in sex differences of hypertension and target organ damage. Biol. Sex Differ..

[127] Cramariuc D., Rogge B.P., Lønnebakken M.T., Boman K., Bahlmann E., Gohlke-Bärwolf C., Chambers J.B., Pedersen T.R., Gerdts E. (2014). Sex differences in cardiovascular outcome during progression of aortic valve stenosis. Heart.

[128] Martínez-Sellés M., Doughty R.N., Poppe K., Whalley G.A., Earle N., Tribouilloy C., McMurray J.J., Swedberg K., Køber L., Berry C. (2012). Gender and survival in patients with heart failure: Interactions with diabetes and aetiology. Results from the MAGGIC individual patient meta analysis. Eur. J. Heart Fail..

[129] Horvath C., Kararigas G. (2022). Sex-dependent mechanisms of cell death modalities in cardiovascular disease. Can. J. Cardiol..

[130] Chang E., Varghese M., Singer K. (2018). Gender and sex differences in adipose tissue. Curr. Diabetes Rep..

[131] Kautzky-Willer A., Harreiter J., Pacini G. (2016). Sex and gender differences in risk, pathophysiology and complications of Type 2 diabetes mellitus. Endocr. Rev..

[132] Mauvais-Jarvis F. (2018). Gender differences in glucose homeostasis and diabetes. Physiol. Behav..

[133] Kanter R., Caballero B. (2012). Global gender disparities in obesity: A Review. Adv. Nutr. Int. Rev. J..

[134] Mauvais-Jarvis F. (2011). Estrogen and androgen receptors: Regulators of fuel homeostasis and emerging targets for diabetes and obesity. Trends Endocrinol. Metab..

[135] Mauvais-Jarvis F., Mauvais-Jarvis F. (2017). Epidemiology of Gender Differences in Diabetes and Obesity. Sex and Gender Factors Affecting Metabolic Homeostasis, Diabetes and Obesity.

[136] Yassin A., Haider A., Haider K.S., Caliber M., Doros G., Saad F., Garvey W.T. (2019). Testosterone therapy in men with hypogonadism prevents progression from prediabetes to type 2 diabetes: Eight-year data from a registry study. Diabetes Care.

[137] Navarro G., Allard C., Xu W., Mauvais-Jarvis F. (2015). The role of androgens in metabolism, obesity, and diabetes in males and females. Obesity.

[138] Power M.L., Schulkin J. (2008). Sex differences in fat storage, fat metabolism, and the health risks from obesity: Possible evolutionary origins. Br. J. Nutr..

[139] Varlamov O., Bethea C.L., Roberts C.T.J. (2015). Sex-specific differences in lipid and glucose metabolism. Front. Endocrinol..

[140] Chaturvedi N., Sjoelie A.-K., Porta M., Aldington S.J., Fuller J.H., Songini M., Kohner E.M. (2001). Markers of insulin resistance are strong risk factors for retinopathy incidence in type 1 diabetes: The eurodiab prospective complications study. Diabetes Care.

[141] Kajiwara A., Miyagawa H., Saruwatari J., Kita A., Sakata M., Kawata Y., Oniki K., Yoshida A., Jinnouchi H., Nakagawa K. (2014). Gender differences in the incidence and progression of diabetic retinopathy among Japanese patients with type 2 diabetes mellitus: A clinic-based retrospective longitudinal study. Diabetes Res. Clin. Pactr..

[142] Schmidl D., Schmetterer L., Garhöfer G., Popa-Cherecheanu A. (2014). Gender differences in ocular blood flow. Curr. Eye Res..

[143] Kazama S., Kazama J.J., Ando N. (2019). Eye diseases in women. Fukushima J. Med Sci..

[144] Sitruk-Ware R. (2016). Hormonal contraception and thrombosis. Fertil. Steril..

[145] Bek T., Lund-Andersen H., Hansen A.B., Johnsen K.B., Sandbaek A., Lauritzen T. (2009). The prevalence of diabetic retinopathy in patients with screen-detected type 2 diabetes in Denmark: The ADDITION study. Acta Ophthalmol..

[146] Foo V., Quah J., Cheung C.M.G., Tan N.-C., Zar K.L.M., Chan C.M., Lamoureux E., Yin W.T., Tan G., Sabanayagam C. (2016). HbA1c, systolic blood pressure variability and diabetic retinopathy in Asian type 2 diabetics. J. Diabetes.

[147] Ding E.L., Song Y., Malik V.S., Liu S. (2006). Sex Differences of endogenous sex hormones and risk of type 2 diabetes: A systematic review and meta-analysis. JAMA.

[148] Coviello A.D., Sam S., Legro R., Dunaif A. (2009). High prevalence of metabolic syndrome in first-degree male relatives of women with polycystic ovary syndrome is related to high rates of obesity. J. Clin. Endocrinol. Metab..

[149] Jayasena C.N., Franks S. (2014). The management of patients with polycystic ovary syndrome. Nat. Rev. Endocrinol..

[150] Hjorth M.F., Roager H.M., Larsen T.M., Poulsen S.K., Licht T.R., Bahl M.I., Zohar Y., Astrup A. (2018). Pre-treatment microbial prevotella-to-bacteroides ratio, determines body fat loss success during a 6-month randomized controlled diet intervention. Int. J. Obes..

[151] Mueller S., Saunier K., Hanisch C., Norin E., Alm L., Midtvedt T., Cresci A., Silvi S., Orpianesi C., Verdenelli M.C. (2006). Differences in fecal microbiota in different European study populations in relation to age, gender, and country: A cross-sectional study. Appl. Environ. Microbiol..

[152] Bridgewater L.C., Zhang C., Wu Y., Hu W., Zhang Q., Wang J., Li S., Zhao L. (2017). Gender-based differences in host behavior and gut microbiota composition in response to high fat diet and stress in a mouse model. Sci. Rep..

[153] Steegenga W.T., Mischke M., Lute C., Boekschoten M.V., Pruis M.G., Lendvai A., Verkade H.J., Boekhorst J., Timmerman H.M., Plösch T. (2014). Sexually dimorphic characteristics of the small intestine and colon of prepubescent C57BL/6 mice. Biol. Sex Differ..

[154] Yurkovetskiy L., Burrows M., Khan A.A., Graham L., Volchkov P., Becker L., Antonopoulos D., Umesaki Y., Chervonsky A.V. (2013). Gender bias in autoimmunity is influenced by microbiota. Immunity.

[155] Shin J.-H., Park Y.-H., Sim M., Kim S.-A., Joung H., Shin D.-M. (2019). Serum level of sex steroid hormone is associated with diversity and profiles of human gut microbiome. Res. Microbiol..

[156] Fransen F., van Beek A.A., Borghuis T., Meijer B., Hugenholtz F., van der Gaast-De Jongh C., Savelkoul H.F., De Jonge M.I., Faas M.M., Boekschoten M.V. (2017). The Impact of gut microbiota on gender-specific differences in immunity. Front. Immunol..

[157] Markle J.G.M., Frank D.N., Mortin-Toth S., Robertson C.E., Feazel L.M., Rolle-Kampczyk U., von Bergen M., McCoy K.D., Macpherson A.J., Danska J.S. (2013). Sex differences in the gut microbiome drive hormone-dependent regulation of autoimmunity. Science.

[158] Acharya K.D., Gao X., Bless E.P., Chen J., Tetel M.J. (2019). Estradiol and high fat diet associate with changes in gut microbiota in female ob/ob mice. Sci. Rep..

[159] Baber R.J., Panay N., Fenton A., the IMS Writing Group (2016). 2016 IMS Recommendations on women’s midlife health and menopause hormone therapy. Climacteric.

[160] Kaliannan K., Robertson R.C., Murphy K., Stanton C., Kang C., Wang B., Hao L., Bhan A.K., Kang J.X. (2018). Estrogen-mediated gut microbiome alterations influence sexual dimorphism in metabolic syndrome in mice. Microbiome.

[161] Atkinson A.J.J., Colburn W.A., DeGruttola V.G., DeMets D.L., Downing G.J., Hoth D.F., Oates J.A., Peck C.C., Spilker B.A., Biomarkers Definitions Working Group (2001). Biomarkers and surrogate endpoints: Preferred definitions and conceptual framework. Clin. Pharmacol. Ther..

[162] Tamhane M., Cabrera-Ghayouri S., Abelian G., Viswanath V. (2019). Review of biomarkers in ocular matrices: Challenges and opportunities. Pharm. Res..

[163] Amitani M., Asakawa A., Amitani H., Inui A. (2013). The role of leptin in the control of insulin-glucose axis. Front. Neurosci..

[164] Laughlin G.A., Barrett-Connor E., Cummins K.M., Daniels L.B., Wassel C.L., Ix J.H. (2013). Sex-specific association of fetuin-a with type 2 diabetes in older community-dwelling adults: The rancho bernardo study. Diabetes Care.

[165] Kadowaki T., Yamauchi T. (2005). Adiponectin and adiponectin receptors. Endocr. Rev..

[166] Li S., Shin H.J., Ding E., van Dam R. (2009). Adiponectin levels and risk of type 2 diabetes: A systematic review and meta-analysis. JAMA.

[167] Chen G.-C., Qin L.-Q., Ye J.-K. (2013). Leptin levels and risk of type 2 diabetes: Gender-specific meta-analysis. Obes. Rev..

[168] Rasul S., Ilhan A., Reiter M.H., Baumgartner-Parzer S., Kautzky-Willer A. (2011). Relations of adiponectin to levels of metabolic parameters and sexual hormones in elderly type 2 diabetic patients. Gend. Med..

[169] Abbasi A., Corpeleijn E., Van Der Schouw Y.T., Stolk R.P., Spijkerman A., Van Der A D.L., Navis G., Bakker S.J.L., Beulens J.W.J. (2012). Parental history of type 2 diabetes and cardiometabolic biomarkers in offspring. Eur. J. Clin. Investig..

[170] Piani F., Melena I., Tommerdahl K.L., Nokoff N., Nelson R.G., Pavkov M.E., van Raalte D.H., Cherney D.Z., Johnson R.J., Nadeau K.J. (2021). Sex-related differences in diabetic kidney disease: A review on the mechanisms and potential therapeutic implications. J. Diabetes its Complicat..

[171] Daniels L.B., Maisel A.S. (2015). Cardiovascular biomarkers and sex: The case for women. Nat. Rev. Cardiol..

[172] Melander O., Maisel A.S., Almgren P., Manjer J., Belting M., Hedblad B., Engström G., Kilger U., Nilsson P., Bergmann A. (2012). Plasma proneurotensin and incidence of diabetes, cardiovascular disease, breast cancer, and mortality. JAMA.

[173] Stadlmayr A., Aigner E., Huber-Schönauer U., Niederseer D., Zwerina J., Husar-Memmer E., Hohla F., Schett G., Patsch W., Datz C. (2014). Relations of vitamin D status, gender and type 2 diabetes in middle-aged Caucasians. Acta Diabetol..

[174] Zorena K., Raczyńska D., Raczyńska K. (2013). Biomarkers in diabetic retinopathy and the therapeutic implications. Mediat. Inflamm..

[175] Kaštelan S., Orešković I., Bišćan F., Kaštelan H., Antunica A.G. (2020). Inflammatory and angiogenic biomarkers in diabetic retinopathy. Biochem. Med..

[176] Ogata N., Matsuoka M., Matsuyama K., Shima C., Tajika A., Nishiyama T., Wada M., Jo N., Higuchi A., Minamino K. (2007). Plasma concentration of pigment epithelium-derived factor in patients with diabetic retinopathy. J. Clin. Endocrinol. Metab..

[177] Ozturk B.T., Bozkurt B., Kerimoglu H., Okka M., Kamis U., Gunduz K. (2009). Effect of serum cytokines and VEGF levels on diabetic retinopathy and macular thickness. Mol. Vis..

[178] Rajab H.A., Baker N.L., Hunt K.J., Klein R., Cleary P.A., Lachin J., Virella G., Lopes-Virella M.F. (2015). The predictive role of markers of Inflammation and endothelial dysfunction on the course of diabetic retinopathy in type 1 diabetes. J. Diabetes its Complicat..

[179] Pusparajah P., Lee L.-H., Kadir K.A. (2016). Molecular markers of diabetic retinopathy: Potential screening tool of the future?. Front. Physiol..

[180] Sharma S., Purohit S., Sharma A., Hopkins D., Steed L., Bode B., Anderson S.W., Caldwell R., She J.-X. (2015). Elevated serum levels of soluble TNF receptors and adhesion molecules are associated with diabetic retinopathy in patients with type-1 diabetes. Mediat. Inflamm..

[181] Midena E., Frizziero L., Midena G., Pilotto E. (2021). Intraocular fluid biomarkers (liquid biopsy) in human diabetic retinopathy. Graefe’s Arch. Clin. Exp. Ophthalmol..

[182] Youngblood H., Robinson R., Sharma A., Sharma S. (2019). Proteomic biomarkers of retinal inflammation in diabetic retinopathy. Int. J. Mol. Sci..

[183] Pang T., Leach S.T., Katz T., Day A.S., Ooi C.Y. (2014). Fecal biomarkers of intestinal health and disease in children. Front. Pediatr..

[184] Sekirov I., Russell S.L., Antunes L.C.M., Finlay B.B., Galla S., Chakraborty S., Cheng X., Yeo J., Mell B., Zhang H. (2010). Gut microbiota in health and disease. Physiol. Rev..

[185] Pedersen H.K., Gudmundsdottir V., Nielsen H.B., Hyotylainen T., Nielsen T., Jensen B.A.H., Forslund K., Hildebrand F., Prifti E., Falony G. (2016). Human gut microbes impact host serum metabolome and insulin sensitivity. Nature.

[186] Si J., Lee C., Ko G. (2017). Oral microbiota: Microbial biomarkers of metabolic syndrome independent of host genetic factors. Front. Cell. Infect. Microbiol..

[187] Rinninella E., Raoul P., Cintoni M., Franceschi F., Miggiano G.A.D., Gasbarrini A., Mele M.C. (2019). What is the healthy gut microbiota composition? A changing ecosystem across age, environment, diet, and diseases. Microorganisms.

[188] Magne F., Gotteland M., Gauthier L., Zazueta A., Pesoa S., Navarrete P., Balamurugan R. (2020). The Firmicutes/Bacteroidetes Ratio: A Relevant Marker of Gut Dysbiosis in Obese Patients?. Nutrients.

[189] Cani P., Delzenne N., Amar J., Burcelin R. (2008). Role of gut microflora in the development of obesity and insulin resistance following high-fat diet feeding. Pathol. Biol..

[190] Bressa C., Bailén-Andrino M., Pérez-Santiago J., González-Soltero R., Pérez M., Montalvo-Lominchar M.G., Maté-Muñoz J.L., Domínguez R., Moreno D., Larrosa M. (2017). Differences in gut microbiota profile between women with active lifestyle and sedentary women. PLoS ONE.

[191] Maric-Bilkan C. (2017). Sex differences in micro- and macro-vascular complications of diabetes mellitus. Clin. Sci..

[192] Krishnan K.C., Vergnes L., Acín-Pérez R., Stiles L., Shum M., Ma L., Mouisel E., Pan C., Moore T.M., Péterfy M. (2021). Sex-specific genetic regulation of adipose mitochondria and metabolic syndrome by Ndufv2. Nat. Metab..

[193] Bennett B.J., Farber C.R., Orozco L., Kang H.M., Ghazalpour A., Siemers N., Neubauer M., Neuhaus I., Yordanova R., Guan B. (2010). A high-resolution association mapping panel for the dissection of complex traits in mice. Genome Res..

[194] Nookaew I., Svensson P.-A., Jacobson P., Jernås M., Taube M., Larsson I., Andersson-Assarsson J., Sjöström L., Froguel P., Walley A. (2013). Adipose tissue resting energy expenditure and expression of genes involved in mitochondrial function are higher in women than in men. J. Clin. Endocrinol. Metab..

[195] Wang W., Lo A.C.Y. (2018). Diabetic retinopathy: Pathophysiology and treatments. Int. J. Mol. Sci..

[196] Iatcu C.O., Steen A., Covasa M. (2021). Gut Microbiota and Complications of Type-2 Diabetes. Nutrients.

[197] Lee C., Chae S., Jo S., Jerng U., Bae S. (2021). The Relationship between the Gut Microbiome and Metformin as a Key for Treating Type 2 Diabetes Mellitus. Int. J. Mol. Sci..

[198] Verma A., Xu K., Du T., Zhu P., Liang Z., Liao S., Zhang J., Raizada M.K., Grant M.B., Li Q. (2019). Expression of Human ACE2 in Lactobacillus and Beneficial Effects in Diabetic Retinopathy in Mice. Mol. Ther. Methods Clin. Dev..

[199] Li Q., Xu K., Du T., Zhu P., Verma A. (2018). Recombinant Probiotics Expressing Angio-tensin-(1-7) Improves Glucose Metabolism and Diabetes-Induced Renal and Retinal Injury. Diabetes.

